# Welfare Assessment and Husbandry Practices of Working Horses in Fiji

**DOI:** 10.3390/ani10030392

**Published:** 2020-02-28

**Authors:** Navina Fröhlich, Patrick D. Sells, Rebecca Sommerville, Charlotte F. Bolwell, Charlotte Cantley, Jessica E. Martin, Stuart J. G. Gordon, Tamsin Coombs

**Affiliations:** 1 The Royal (Dick) School of Veterinary Studies, The College of Medicine and Veterinary Medicine, Easter Bush Campus, The University of Edinburgh, Edinburgh EH25 9RG, UK; 2 Chasemore Farm, Cobham, Surrey KT11 3JT, UK; pat@chasemorefarm.co.uk; 3 Brooke, 2nd Floor, The Hallmark Building, 52–56 Leadenhall Street, London EC3M 5JE, UK; Becca-S@hotmail.co.uk; 4 School of Veterinary Science, Massey University, Tennent Drive, Palmerston North 4474, New Zealand; C.Bolwell@massey.ac.nz (C.F.B.); S.J.G.Gordon@massey.ac.nz (S.J.G.G.); 5 New Zealand Veterinary Association, Level 2, 44 Victoria Street, Wellington 6011, New Zealand; charlotte.cantley@vets.org.nz; 6 The Royal (Dick) School of Veterinary Studies and The Roslin Institute, The College of Medicine and Veterinary Medicine, Easter Bush Campus, The University of Edinburgh, Edinburgh EH25 9RG, UK; 7 Animal & Veterinary Sciences, Scotland’s Rural College (SRUC), Peter Wilson Building, Kings Buildings, West Mains Road, Edinburgh EH9 3JG, UK; Tamsin.Coombs@sruc.ac.uk

**Keywords:** Fiji, working horse, welfare, intervention, husbandry, healthcare

## Abstract

**Simple Summary:**

In low and middle-income countries, working equids play an essential role in supporting the livelihoods of their owners. The primary objective of the study was to provide the first description of the welfare status of working horses in Fiji by analysing owner and animal-based parameters and owner perceptions of husbandry and healthcare. A total of 279 Fijian horse owners were questioned on their husbandry and healthcare practices and the welfare of their horses was assessed. Horse owners who were supporting a greater number of dependent family members had horses with an increased prevalence of wounds than those with less dependents. Wounds were more prevalent in horses used for draught work and “carrying people or goods on back” while hoof neglect was associated with draught and breeding/other work. A lower body condition score was found in horses with neglected hooves and the presence of hoof neglect and wounds was associated with a negative general attitude in these horses. However, this study also found indicators of good welfare in these horses. These findings suggest that intervention, in the form of targeted veterinary services alongside training programs for owners, is required in order to improve the welfare of working horses in Fiji.

**Abstract:**

Research shows that working equids in low and middle-income countries play an essential role in supporting the livelihoods of their owners. The objective of the study was to provide the first description of the welfare status of working horses in Fiji by analysing animal-based parameters alongside owner knowledge and perceptions of horse management. Trained assessors used a structured interview to question 279 horse owners on their knowledge and management practices, while their horses (*n* = 672) were assessed on health and welfare parameters. Horse owners supporting five or more dependent family members had horses with an increased prevalence of wounds than those with less dependents. The presence of wounds was associated with draught work and “carrying people or goods on back” while hoof neglect was associated with draught and breeding/other work. A lower body condition score was found in horses with neglected hooves and the presence of hoof neglect and wounds was associated with a negative general attitude in these horses. However, this study also found indicators of good welfare in these horses. These findings suggest that intervention, in the form of targeted veterinary services alongside training programs for owners, is required in order to improve the welfare of working horses in Fiji.

## 1. Introduction

Working animals are an essential resource and are vital to the income, workload, social and cultural life of their owners. Equids are the second-most commonly used working animal [1] with approximately 100 million working horses, mules and donkeys in low- and middle-income countries (LMIC) [2]. Some owners in LMIC countries, who depend on animals for their livelihood [3], are unable to provide their equids with adequate nutrition and water, shelter, appropriate tack or veterinary care and rely on the services of non-governmental organisations (NGOs). These NGOs train veterinary professionals and support equine welfare interventions for communities in LMIC. The most commonly reported health issues in working equids are wounds and lesions, poor body condition, gait abnormalities and diseases [4,5,6,7]. A high prevalence of pain and fear, as indicated by behaviour, have been associated with physical health problems [6,8]. These issues can lead to compromised welfare, productivity and longevity in working equids, which may affect owners economically and further exacerbate the poverty cycle [6]. 

Despite their high socio-economic importance, working equids are often not included in agricultural and animal health policies, education systems and research [9]. The working equid population in Fiji, mainly consisting of horses [10], has not been investigated to date. Horses are essential for transportation in Fiji because of the steep and mountainous terrain, and the secluded farming communities. The Food and Agriculture Organisation named inbreeding, inadequate nutrition and husbandry as the causes of limited productivity of equids in the South Pacific Region [3]. Three of the co-authors, working with the Pacific Education and Animal Trust (a New Zealand-based working animal charitable trust) in Fiji identified poor management, specifically the use of inappropriate saddles on horses, associated with a poor body condition score (BCS), and wounds on the back, as a welfare concern. Based on these observations, formal research was undertaken to investigate the welfare of working horses in Fiji.

The objectives of this study were (1) to provide the first description of the welfare status of the working horse population in Fiji by analysing owner and animal-based parameters and owner perceptions of husbandry and healthcare and (2) to investigate the relationships between these parameters in order to identify potential risk factors for poor equine welfare. The results will contribute to the development of specific strategies to improve working equine welfare in Fiji.

## 2. Materials and Methods 

Data were collected between June 2015 and June 2016. Villages were selected incidentally, either due to their proximity to an agricultural extension project (convenience sampling), or during visits to villages with known horse populations in need of direct service provision (purposive sampling) in Fiji. Mostly, these were isolated communities in Fiji’s mountainous interior and were located in 11 districts of four different regions of Viti Levu. In total, 343 horse owners were interviewed by two trained assessors. Owners were included in the study if they owned at least one horse and were willing to participate. Owners were interviewed at home in each village and their horses were assessed, if available and present during the visit. Global positioning system (GPS) co-ordinates were taken and recorded using a handheld GPS unit (Garmin Etrex 20x, Garmin Ltd., Olathe, Kansas, USA) in order to accurately identify villages and to allow for the spatial analysis of data. The assessors communicated with the horse owners using the local Fijian dialect. The interview data were then entered into the owner questionnaire forms in English. 

### 2.1. Owner Questionnaire

The owner questionnaire (Table S1, Supplementary Material) was divided into five sections: location (province, district, village, GPS coordinates) and general information (name, gender, age, ethnicity, number of dependents, type of farm, type of lease), number of types of livestock owned (horses, cows, chicken, goats, sheep and pigs), husbandry, watering (constant access to water and frequency of water provision) and, lastly, healthcare. “Number of dependents in family” was included as a novel parameter, with dependents being defined as people who rely on the income and resources of the horse owner. The husbandry section included information about the frequency of deworming, dental care and hoof care and who it was done by, as well as the castration age of horses, the technique used, who it was done by and the reasons for the castration. The healthcare section included questions on the expected lifespan of a horse, at what age a horse starts to be ridden, who carried out its healthcare, how long the owner had been working with horses, how many of their horses had died and what the causes were, as well as what owners do when their horses reach the end of their working lives. Furthermore, this section contained questions about important health problems in horses and cattle and how they are solved, knowledge about and experiences with locally active animal welfare organisations, required services (including willingness to pay for them) and educational workshops and, lastly, the use of medical plants. Horse owners were then asked to point out any welfare concerns relating to their horses and those topics that they felt they needed help with and/or further education.

### 2.2. Horse Form

A total of 708 horses were assessed. Horses were assessed in their normal working environment by one assessor. Each horse was considered individually, either within its usual working group or while separated. Tack was removed for assessment. A horse form (Table S2, Supplementary Material) was used to record data. The form included descriptive information (name of horse owner, ID, colour, age and sex of horse), information about work (what type of work they were used for, number of hours of work per day, number of days of work per week), health parameters (BCS, signs of hoof neglect, lip lesions, the presence/absence, location and number of swollen joints, the number of wounds as well as a score, surface area and presence/absence of signs of infection), general attitude and the type of bit used. Horses were assessed by observation only and were not touched by the assessor. Handling (e.g., the lifting of hooves) was done by the owner. Hooves were considered neglected if they were overgrown, cracked, deformed and/or showed signs of white line disease, abscesses and thrush. Wounds were recorded on an equine dermatological map. They were numbered and scored objectively according to their severity and their surface area measured. BCS was assessed using a nine-point system, as adopted by the American Association of Equine Practitioners Equine Welfare Committee [11]. Scoring systems for lip lesions and general attitude were taken from the Standardised Equine-Based Welfare Assessment Tool [12]. Lip lesions were analysed by looking at the corners (commissures) of the lips on both sides and given scores from zero (no lesions present) to three (deep lesions). General attitude was evaluated by considering the behaviour of the horse throughout the whole assessment (how it reacted to the surroundings, the handler, the assessor, being handled) and was scored from zero to two (0 = positive general attitude: bright, alert, responsive, 1 = negative non-reactive general attitude: dull, obtund, lethargic, and 2 = negative reactive general attitude: fearful, aggressive, signs of anxiety). “Traumatic” metal bits were defined as those with sharp, rough or abrasive edges capable of damaging the soft tissues of the mouth (e.g., sharp metal piping), whereas “humane” metal bits were defined as those that were smooth and atraumatic.

### 2.3. Ethical Review

A Low Risk Notification was approved on 11 May 2015 by the Massey University Human Ethics Committee. Horse owners were informed of the purpose of this study. Verbal informed consent was obtained from all owners participating in the study.

### 2.4. Statistical Analysis

The data of only 279 horse owners and their 672 horses were used for analysis. Due to unrecorded information, the remaining horses that were assessed (36) could not be matched with the remaining owners that were interviewed (64). For a number of parameters from the owner questionnaire (number of dependents, experience with horses in years, number of horses and other livestock animals, horse starting age, horse lifespan) and the horse form (horse age, horse workload in days per week and hours per day), the raw data were reclassified as ordinal groups in order to enable analysis. In some instances, variables were re-categorised in order to reduce the sparsity of data in certain categories. Horse use was re-categorised from 11 different work types to three broad categories of work (draught, goods/people carried on back and breeding/other). General attitude was re-categorised from three nominal levels of positive, negative non-reactive and negative reactive, to binary levels of positive or negative behaviour (negative non-reactive and negative reactive). See Table 1 for details of this re-categorisation. All data were summarised and analysed using Genstat (18th Edition, VSN International, Hemel Hempstead, UK). Statistical comparisons were conducted via Generalised Linear Mixed Models (GLMM), using Residual Maximum Likelihood (REML) in Genstat. Poisson, nominal or binomial distributions were used depending on the response variable investigated. Dispersion was fixed at one for all models. All models had observer and owner ID as random effects, in order to account for multiple horse ownership and observer variations. Data transformations were attempted when necessary via the logarithm or logit function. All fixed effects were treated as factors and all interactions between factors were included in maximal models. Statistical significance was based on approximate F tests, when these were available, comparing F statistics to the F distribution and *p* < 0.05 significance level. All models included owner demographic data (e.g., ethnicity, gender and farm) as well as horse parameters (e.g., horse age group, type of bit, type of work and workload) as fixed effects. The total number of livestock was included as a co-variate in all models. Welfare indicators (e.g., wounds, BCS, hoof neglect, lip lesions) were also included as fixed effects in order to obtain an association with the response variable welfare indicator. Modelling was not conducted on those data variables with insufficient data (e.g., lip lesions).

For most parameters, there were a number of missing values (mean [± SE] % of missing data per variable = 19.7 ± 0.7%; range = 0–83%) due to horse owners not wanting, or not being able, to answer particular questions. The range of missing data varied greatly depending on the section (owner information: mean [± SE] = 17.7 ± 1.9%; owner reported management practices: mean [± SE] = 41.8 ± 1.9%; horse information: mean [± SE] = 3.8 ± 0.1%). All percentages were therefore calculated from the available data for each parameter, excluding missing values. 

## 3. Results

### 3.1. Owner Information

Horse owners were predominantly male (186, 89.0%) and of Fijian ethnicity (150, 71.8%). A total of 177 (67.1%) horse owners had three or more dependent family members, with only 46 (17.4%) having no dependents. The average age of owners was 37.7 years old (SD = 16.2), although 17 owners (6.7%) were less than 16 years old. Most owners had at least 10 years of experience in caring for horses (148, 89.2%) and also reported having less than five horses (214, 77.3%), with only five people (1.8%) owning more than 10 horses. Just under half (120, 43.0%) owned 10 or more livestock animals (chickens, cattle, sheep, goats or pigs), while 59 people (21.2%) owned no livestock at all, except horses. Most farms (116, 45.1%) were reported as pastoral, with 81 (31.5%) reported as arable. Over half of these farms were leased formally (143, 57.9%). See Table 2 for a full description of this data.

### 3.2. Owner Reported Management Practices

When asked about horse management practices, most owners reported that their horses did not receive anthelmintic treatment (238, 95.2%) or hoof care (235, 94.0%). Furthermore, only four owners (8.5%) reported that their horses received dental treatment. More than half of owners (89, 62.7%) said that they provided healthcare for their horses themselves while most (158, 87.3%) reported that their horses had constant access to water. Most owners (147, 96.1%) also thought that horses should begin to work between three and five years of age and 115 owners (67.7%) stated that they believed that horses generally live for more than 30 years. Most owners (118, 88.7%) reported that they had experienced three or less deaths of horses in their care, with the main reasons for these deaths being accident (36, 30.5%), old age (25, 21.2%), medical issues (23, 19.5%) and natural disasters (20, 16.9%). However, 11 (9.3%) owners reported that their horses had died as a result of strangulation. Furthermore, most owners (160, 95.8%) stated that they abandoned their horses once the horses were no longer able to work. Over half of owners (91, 54.5%) reported that there were no particular difficulties that hampered their ability to care for their horses. However, 49 (29.3%) reported that drought was a significant issue. See Table 3 for a full description of this data.

### 3.3. Horse Information

Data were collected from 672 horses, with most being in the Ba (547, 81.4%) and Nadrogra/Navosa (115, 17.1%) regions of Viti Levu. Of these, 228 (34.9%) horses were less than 6 years old and 394 (60.3%) horses were aged between 6 and 15 years. Just over half (398, 59.7%) of these horses were colts or stallions, with a further 214 (32.1%) mares and the remaining 40 horses (6.0%) geldings. They performed a number of roles with most (209, 34.8%) classified as multipurpose working animals which were used for a variety of purposes such as transport, riding, agriculture, hunting, fishing or racing. Very few horses (54, 8.5%) did no work at all, while 347 (54.9%) worked less than 5 h per day. Most horses (422, 69%) worked between four and seven days per week. When worked, most horses were bitted with a metal bit with 236 (35.8%) of these horses bitted with a “humane” metal bit and 261 (39.6%) with a “traumatic” metal bit. Most horses (601, 90.4%) had a BCS ≥ 4 (moderately thin) on a scale of 1–9 (1 = poor; 9 = extremely fat) and 469 (71.5%) showed signs of hoof neglect. Of the other welfare parameters, lip lesions were seen in 223 (34.4%) of horses, 144 (22.2%) had other wounds and 27 (4.1%) had swollen joints. General attitude was recorded as positive in most (579, 89.2%) horses. See Table 4 for a full description of this data.

### 3.4. Relationship between Owner-Based Parameters and Animal-Based Outcome Measures

The number of human dependents being supported by a horse owner was associated with the presence of wounds (F = 9.92_(2,651)_, *p* < 0.001), with horses belonging to owners who were supporting five or more dependent people showing a higher occurrence of wounds than those with less dependents. The type of work was also associated with the presence of wounds, with horses being used for draught purposes (F = 6.70_(1,651)_, *p* = 0.010) and “carrying people or goods on back” (F = 6.82_(1,651)_, *p* = 0.009) showing a higher prevalence of wounds compared to horses who did not perform these methods of work. There was no relationship between performing breeding/otherwork on the presence of wounds. Owner gender, farm type, type of bit, number of working days per week and the total number of livestock owned had no relationship with the prevalence of wounds. 

There was a weak tendency for horse owners who leased mixed farms to have more horses showing signs of hoof neglect than those who leased arable or pastoral farms (F = 2.74_(1,651)_, *p* = 0.099). Horses bitted with “humane” metal bits had a lower prevalence of hoof neglect than those bitted with all other bit types (F = 3.29_(4,648)_, *p* = 0.011). Overall, work type category was associated with hoof neglect, with a greater occurrence in work types draught (F = 5.15_(1,651)_, *p* = 0.024) and breeding/other (F = 3.35_(1,651)_, *p* = 0.068). However, there was no association between hoof neglect and whether the horses were used for “carrying people or goods on back”. The total number of livestock, the gender of the owner, the number of working days per week and the number of dependents were not associated with the presence of hoof neglect.

BCS was associated with the type of bit used (F = 7.13_(4,648)_, *p* < 0.001), with marginally lower BCS in animals bitted with “traumatic” metal and no bits compared to “humane” metal, rope, and other bits. There was no relationship between the category of work the horses performed and BCS. The remaining factors: ethnicity, gender, farm type, the number of working days per week and the number of dependents were also not related to BCS. 

The general attitude of the horses was not associated with any owner parameters: ethnicity, total number of livestock, owner gender, farm type, number of dependents, type of work and type of bit. See Table 5 for full results of all significant associations.

### 3.5. Relationship between Animal-Based Parameters and Animal-Based Outcome Measures

Older horses showed a higher prevalence of hoof neglect when compared to younger horses (F = 5.30_(2,650)_, *p* = 0.005)). There was also an association between general attitude and hoof neglect, with horses showing negative behaviour having a higher prevalence of hoof neglect when compared to those showing positive behaviour (F = 7.31_(1,651)_, *p* = 0.007). There was no association between the presence of lip lesions and the presence of hoof neglect. 

There was a tendency towards an association between general attitude and the presence of wounds (F = 3.44_(1,651)_, *p* = 0.064), with horses showing negative behaviour having a higher prevalence when compared to horses showing positive behaviour. Horses with no hoof neglect were significantly more likely to have a higher occurrence of wounds (F = 4.30_(1,651)_, *p* = 0.039), compared to horses with hoof neglect. There was no relationship between BCS, the presence of lip lesions, and the presence of wounds. 

Only hoof neglect was associated with BCS (F = 7.83_(1,651)_, *p* = 0.005), with higher BCS associated with the absence of hoof neglect. However, the interpretation of this association should be considered carefully, as there was a heavy bias of horses from the population falling in the BCS ranges of less than five.

Horse age, the presence of lip lesions or wounds and the general attitude of the horses had no relationship with BCS. There was no association between the general attitude of horses and any of the following parameters: lip lesions, horse age and BCS. See Table 6 for full results of all significant associations.

## 4. Discussion

The aim of this study was to investigate the welfare status of working horses in Fiji by analysing owner and animal-based parameters and their relationships and owner perceptions of husbandry and healthcare. The data analysis identifies risk factors for poor welfare and provides a first indication of the welfare status.

The results showed that just over half of the horses were intact males with very few geldings, which is supported by other studies on working equid welfare where veterinary provision is limited and where castration could carry a significant risk to welfare [4,7]. That there is a lack of professional veterinary and hoof care provision in Fiji is further supported by the results from the owner questionnaire, which showed that only a very small number of horses were provided with hoof care, dental care and anthelmintic treatment and even fewer with professional veterinary care. Medical issues were also cited as being one of the main causes of death of horses in their care. 

The analysis showed that most participating owners “let their horses go” once the horses could no longer work. This is the process of abandoning the horse and terminating all feeding and husbandry obligations. Pritchard et al. [13] stated that euthanasia of working equines is rarely an option in LMIC due to cultural attitudes, a lack of trained personnel, a lack of availability of appropriate methods and financial reasons, resulting in abandonment and a prolonged, painful death. It is likely that owners “let their horses go” when these horses are old and in poor condition at the end of their working life and they may ultimately starve to death or be fatally struck by vehicles while wandering near major roads. It is interesting to note that the main cause of death of horses cited by owners was “accident”, which may relate to incidents after such an abandonment. Unfortunately, it is unclear from the owner responses as to when these “accidents” took place. However, the abandonment of horses is clearly a significant welfare concern in Fiji and requires further investigation. Although only a small number of owners named strangulation by rope a reason of death, it might be more common than implied, as there are only a few alternatives besides abandonment. Furthermore, owners might be aware of the technique being an extreme use to euthanize an animal and, therefore, adjusted their answers to the assessors’ opinions or wishes (the Hawthorne effect). Upjohn et al. [14] reported that horse owners in Lesotho tended to adapt their answers to what the person carrying out the survey wanted to hear or what they believed would result in personal benefits. 

According to horse owners, natural disasters are also a common cause of death for horses. Furthermore, they also stated that droughts are a threat to their ability to care for an animal. Despite the potential threat from droughts, and flooding which can occur in the rainy season (from November to April), no disaster management protocol for working animals is in place in Fiji (Ricketts, personal communication). Including animals in disaster management protocols is important for both animal welfare and the protection of the livelihoods of animal owners [15]. Other LMIC with relevant protocols are Mexico, Chile and Guatemala [16].

This study is the first to investigate the relationship between the number of dependents and working equid welfare parameters. This is relevant given the livelihood benefits of working equids within human communities. Wounds were found in less than a quarter of the assessed population, which demonstrates a lower prevalence than that found by Luna et al. [17] where 47% of the assessed Chilean draught horse population had skin lesions. However, wounds were more frequent if owners had five or more dependents. Working animals are recognised as being an important source of income for families in LMIC [3]. Therefore, a higher number of dependents may result in a higher workload for each horse to meet the demands of more people. Upjohn [14] found tack to often be inappropriate, so an increased workload due to an increased number of dependents might result in longer exposure to poorly fitting equipment and wounds. Further investigation is needed to test the value of the parameter “number of dependents” for equid welfare assessments in LMIC because the parameter did not appear to be related to any other animal-based outcome parameter. 

In this study, 90.4% of animals had a BCS of four or higher. Pritchard et al. [4] reported a much higher prevalence of thin horses in Afghanistan, Egypt, India, Jordan and Pakistan using a 1–5 scale (69.8% of horses scored 2 or less; 1 = very thin, 5 = very fat) which suggests that working horses in Fiji are generally in good bodily condition in contrast to equids working in certain other LMIC. In this study, a lower BCS was associated with the presence of hoof neglect. A low BCS and presence of hoof neglect might be signs of insufficient care by the owner, which may be as a result of a lack of knowledge or a lack of resources to be able to care for the horse. Tadich et al. [18] found food and hoof management to be the most prominent concerns in a population of Chilean draught horses, and suggested these findings were due to a lack of education and perception of health and husbandry issues. Previous studies on working equid welfare [4] have also investigated lameness alongside hoof neglect. Lameness is a prominent health issue in working horses in LMIC and has been associated with pain [19]. The results from our study showed that hoof neglect was associated with negative general attitude, which suggests that horses with neglected hooves may have been in pain. Pain is a major welfare concern and likely to cause loss of appetite [20], which may result in a lower BCS. 

Increased signs of hoof neglect were found in older horses, which agrees with the findings of Burn et al. [6]. This may be due to reduced productivity towards the end of their working lifespan, leading to neglect from the owner. The results also showed an association between hoof neglect and the presence of wounds. Horses with no hoof neglect were more likely to have a higher occurrence of wounds. These findings may relate to the amount and type of work that animal was doing and the surfaces that they were working on. As discussed previously, those horses that were thought to be working “hardest” were found to have more wounds and it is conceivable that increased movement over abrasive surfaces (because of this increased workload) may result in increased wear on the hooves. This is further supported by the observation that although only 15 owners said that their horses received any kind of hoof care, 187 horses showed no signs of hoof neglect, which suggests some kind of self-trimming resulting from movement. Increased signs of hoof neglect were also found to be related to use for breeding and other work. In this study, it was observed that breeding stock were generally turned out with little human contact, resulting in inadequate care, and were not necessarily undertaking significant movement over abrasive surfaces for work purposes, which may explain the increased occurrence of hoof neglect in those horses. Overall, the occurrence of hoof neglect was found to be higher than in comparable studies. Pritchard et al. [4] reported 55.5% of examined horses to have overgrown hooves. Upjohn [14] found 39.8% (hindfeet) to 44.6% (forefeet) of horses to have overgrown hooves and 9.8% (hindfoot) to 21.3% (forefoot) had foot injuries. This finding may relate to a lack of hoof care provision with only 6% of owners stating that they provided this. This increased prevalence of hoof neglect may also relate to decreased workload, differences in the type of work and differences in the surfaces that the animals are moving over when compared to other studies. In this study more than half of the horses worked less than 5 h a day and over a third worked between 5 and 7 h, which is less than other studies suggest for comparable populations [21]. Further investigation is required in order to fully understand the reasons for such high levels of hoof neglect and as this constitutes a potentially significant welfare issue, provision of appropriate farrier services and training and/or resources in order to enable owners to maintain their horse’s hooves would be beneficial. 

Most horses had a positive general attitude, which agrees with previous studies on the attitude of working equids towards humans [13,18]. Those horses reported with a negative general attitude were more likely to have neglected hooves and there was a tendency for them to have more wounds. Similar studies have found a relationship between a negative general attitude in horses and various welfare parameters [4,8,22]. These findings lend support to behaviour being a useful measure of welfare in working equids as it may be a more direct indicator of how welfare issues are making an animal feel [6]. In future studies, it would be beneficial to include more behavioural assessment parameters in order to help understand how an animal is being affected by specific welfare issues. Although only a small number of animals were reported as negative non-reactive (e.g., apathetic) which is in agreement with [6,8], a non-reactive apathetic response is often seen as normal and not recognised as a risk to horse welfare [6]. It is then not recorded as a negative behaviour which results in under-reporting during welfare assessment. Apathy in working equids requires further investigation in order to understand it underlying causes, refine its definition and, therefore, improve its recognition. Working equids in an apathetic state may be in pain, suffering from exhaustion or other conditions, such as heat stress [6]. They may also be suffering from learned helplessness which can be described as a depressive like state whereby an animal has learnt that it has no control over unpleasant or harmful conditions, and, therefore, “gives up” and no longer reacts to stimuli, aversive or otherwise [23]. Such animals may just be seen as lazy [21] and treated accordingly by their owners which is clearly a significant welfare issue that requires further address through research, and most importantly by those working within communities to improve equid welfare.

The most commonly reported type of bit was the “traumatic” metal bit, which was defined as a bit with sharp, rough or abrasive edges capable of damaging the soft tissues of the mouth. Horses bitted with “humane” metal bits showed less signs of hoof neglect than those bitted with “traumatic” metal bits, rope or no bits. The use of a “humane” bit might be an indicator for a better attitude of horse owners towards their animal’s welfare, better general care or the availability of resources to afford better tack. Interestingly horses bitted with “traumatic” metal bits and no bits had a marginally lower BCS, which might be indicative of the converse to the above result. It would be beneficial to investigate what additional types of bits were used because, compared to metal bits, the use of “other” bits was related to the lowest occurrence of hoof neglect. However, this result should be approached with caution, as there were only eight horses where an “other” bit was used and it is likely that there was considerable variation in what that “other” bit was made from, making it difficult to draw any firm conclusions from this. It would also be beneficial to investigate if there is an association between lip lesions and a specific type of bit. Lip lesions were found in roughly one third of the assessed population (in agreement with Pritchard et al. [4], 31.9%) and could not be investigated as a response variable. The parameter was used as a direct welfare indicator by other studies [4,8] and was likely to be caused by ill-fitting or improper bits or potentially rough handling. 

Analysis also found an increased number of wounds in horses used for carrying people or goods on their backs. It was observed by the assessors in this study that many horses were ridden using a folded hessian sack, an inadequate/damaged saddle, or bareback, and the riding position was over the thoracolumbar spine rather than the (Western) traditional region immediately behind the withers. These practices may be contributory factors to wound formation in this population. Draught horses were found to have an increased number of wounds and neglected hooves. Pritchard et al. [4] stated that draught horses pull heavy loads for a long time daily and found them to have lesions in different body areas and gait abnormalities. However, the results of this study showed that more than half of the horses worked less than 5 h a day and over a third worked between 5 and 7 h, which is less than other studies suggest for comparable populations [21]. It should be noted that most horses were used for more than one type of work, which might confound some of the findings. A more detailed investigation of the different work types undertaken by horses in Fiji is, therefore, needed to establish their effects on working horse welfare. 

Most horse owners did not answer all questions from the questionnaire during the interview and data were collected by convenience and purposive sampling. Taking this and the Hawthorne effect into account, the findings of the interview element of the study may be biased and, therefore, must be viewed with caution. 

In conclusion, signs of good welfare in the Fijian horse population were found with most horses being of moderately thin or above BCS, being free from wounds and reported as having a positive general attitude. However, there was a high occurrence of hoof neglect, a greater occurrence of wounds in horses used for specific types of work and evidence of potentially significant welfare issues when horses reach the end of their working life. These findings indicate that intervention in the form of targeted provision of veterinary and hoof care services alongside participatory workshops and training programs for owners is recommended in order to improve the welfare of working horses in Fiji. Going forward, a better understanding of what factors influence horse owners’ behaviour, for example, social and cultural influences, will help guide these interventions. This study was the first to include the novel parameters “number of dependents” and “type of bit” in the welfare assessment and found significant associations with at least one animal-based welfare parameter. This suggests that both are valuable parameters for use within working equid welfare assessments and should be considered for future research. 

## Figures and Tables

**Table 1:** Grouping and re-categorisation of data for analysis purposes.

Owner Questionnaire		
Group	Coding	Description
Age of owner	1	<16 years
	2	16–24 years
	3	25–35 years
	4	36–50 years
	5	>50 years
Number of dependent people in family	0	0 people
	1	1–2 people
	2	3–4 people
	3	>4 people
Length of time working with horses	1	<10 years
	2	10–20 years
	3	>20 years
Number of horses owned	1	<5 horses
	2	5–10 horses
	3	>10 horses
Number of livestock animals owned	0	No livestock
	1	<10 animals
	2	10–20 animals
	3	>20 animals
Age that horses begin working	1	<3 years
	2	3–5 years
Horse lifespan	1	<20 years
	2	20–30 years
	3	>30 years
Horse Form		
Group	Coding	Description
Age of horse	1	<6 years
	2	6–15 years
	3	>15 years
Horse workload in hours per day	0	0 h
	1	<5 h
	2	5–7 h
Horse workload in days per week	0	0 days per week
	1	1–3 days
	2	4–5 days
	3	6–7 days
Horse work type category	1	Used for draught work (transport of goods and people by cart; agriculture)
	2	Carrying goods or people on back (transport of goods by pack; agriculture; riding animals: transport, tourism, racing, hunting, fishing)
	3	Used for breeding/other work
General Attitude	0	Positive behaviour (Score 0)
	1	Negative behaviour—non-reactive (Score 1) or reactive (Score 2)

**Table 2:** Summary of owner information as collected during interviews. The total sample size for each parameter is in brackets.

Owner Information	Level	*n*	%
Gender (*n* = 209)	Male	186	89.0
	Female	23	11.0
Ethnicity (*n* = 209)	Fijian	150	71.8
	Indian Fijian	58	27.8
	Other	1	0.5
Age (*n* = 255)	<16 years	17	6.7
	16–24 years	39	15.3
	25–35 years	65	25.5
	36–50 years	81	31.8
	>50 years	53	20.8
Number of dependent people (*n* =264)	0 people	46	17.4
	1–2 people	41	15.5
	3–4 people	123	46.6
	>4 people	54	20.5
Length of time working with horses (*n* = 166)	<10 years	18	10.9
	10–20 years	107	64.5
	>20 years	41	24.7
Number of horses owned (*n* = 277)	<5 horses	214	77.3
	5–10 horses	58	20.9
	>10 horses	5	1.8
Number of livestock animals owned (*n* = 279)	No livestock	59	21.2
	<10 animals	100	35.8
	10–20 animals	58	20.8
	>20 animals	62	22.2
Type of farm (*n* = 257)	Pastoral	116	45.1
	Arable	81	31.5
	Mixed	58	22.6
	Other	2	0.8
Type of lease (*n* = 247)	Formal	143	57.9
	Informal	104	42.1

**Table 3:** Summary of management practices as reported by owners during interviews. The total sample size for each parameter is in brackets.

Owner Reported Management Practices	Level	*n*	%
Horses receive anthelmintic treatment (*n* = 250)	Yes	12	4.8
	No	238	95.2
Horses receive hoof care (*n* = 250)	Yes	15	6.0
	No	235	94.0
Horses receive dental treatment (*n* = 47)	Yes	4	8.5
	No	43	91.5
Provider of healthcare to horses (*n* = 142)	Owner	89	62.7
	Vet	2	1.4
	Other person	8	5.6
	No healthcare provision	43	30.3
Horses have constant access to water (*n* = 181)	Yes	158	87.3
	No	23	12.7
Age that horses begin working (*n* = 153)	<3 years	6	3.9
	3–5 years	147	96.1
Horse lifespan (*n* = 170)	<20 years	17	10.0
	20–30 years	38	22.3
	>30 years	115	67.7
Number of horse deaths (*n* = 133)	0 horses	32	24.1
	1–3 horses	86	64.7
	>3 horses	15	11.2
Cause of death (*n* = 118)	Old age	25	21.2
	Accident	36	30.5
	Natural disaster	20	16.9
	Medical issues	23	19.5
	Strangled	11	9.3
	Other reasons	3	2.5
End of working life provision for horses (*n* = 167)	Abandonment	160	95.8
	Other	7	4.2
Issues which make it difficult to care for horses (*n* = 167)	No issues	91	54.5
	Drought	49	29.3
	Equipment	5	3.0
	Health	15	9.0
	Nature	2	1.2
	Other	5	3.0

**Table 4:** Summary of horse information obtained during welfare assessments. The total sample size for each parameter is in brackets.

Horse Information	Level	n	%
Location in Vitu Levu (*n* = 672)	Ba	547	81.4
	Nadrogra/Navosa	115	17.1
	Central	9	1.3
	Other	1	0.2
Age (*n* = 653)	<6 years	228	34.9
	6–15 years	394	60.3
	>15 years	30	4.6
Sex (*n* = 667)	Colt or stallion	398	59.7
	Mare	214	32.1
	Gelding	40	6.0
Type of work (*n* = 620)	Single work purpose	216	34.8
	Multipurpose	209	33.7
	Multipurpose and breeding	158	25.5
	Breeding	37	6.0
Horse workload in hours per day (*n* = 633)	0 h	54	8.5
	<5 h	347	54.9
	5–7 h	232	36.7
Horse workload in days per week (*n* = 612)	0 days	52	8.5
	1–3 days	138	22.6
	4–5 days	311	50.8
	6–7 days	111	18.1
Type of bit (*n* = 659)	“Humane” metal bit	236	35.8
	“Traumatic” metal bit	261	39.6
	Rope bit	65	9.9
	Other bit	8	1.2
	No bit	89	13.5
BCS (*n* = 665)	Score 1	4	0.5
	Score 2	9	1.4
	Score 3	51	7.7
	Score 4	443	66.6
	Score 5	136	20.5
	Score 6	10	1.5
	Score 7	9	1.4
	Score 8	3	0.4
	Score 9	0	0.0
Presence of hoof neglect (*n* = 656)	Yes	469	71.5
	No	187	28.5
Presence of lip lesions (*n* = 649)	Yes	223	34.4
	No	426	65.6
Presence of other wounds (*n* = 649)	Yes	144	22.2
	No	505	77.8
Presence of swollen joints (*n* = 666)	Yes	27	4.1
	No	639	95.9
General attitude (*n* = 649)	Score 0: Positive	579	89.2
	Score 1: Negative un-reactive	49	7.6
	Score 2: Negative reactive	21	3.2

**Table 5:** Results of Generalised Linear Mixed Models (GLMM) models testing showing relationships between owner-based parameters and animal-based outcome measures. Only tendencies (*p* <0.01) or significant effects (*p* < 0.05 in bold) are presented here. The parameter levels where no data was available (e.g., reference level for odds ratio) were identified with an asterisk (*). The means (± standard error of the means), medians, interqurtile ranges (IQR), odds ratio (including associated 95% confidence intervals), F statistics and *p*-values are reported at parameter level.

Animal-Based Outcome Measures	Owner-Based Parameters	Level	Mean (± SE)	Median (IQR)	Odds Ratio (95% CI)	F st	*p*-Value
Presence of wounds	Number of dependent people	0 people	0.23 ± 0.05	0.0 (0.0)	*	9.92	**<0.001**
		1–2 people	0.03 ± 0.02	0.0 (0.0)	0.43 (0.20,0.92)		
		3–4 people	0.09 ± 0.03	0.0 (1.0)	1.18 (0.60,2.31)		
		>4 people	0.25 ± 0.07	1.0 (1.0)	3.812 (1.64,8.86)		
	Used for draught work	Yes	0.58 ± 0.01	1.0 (1.0)	5.81 (3.41,9.87)	6.70	**0.010**
		No	0.19 ± 0.02	0.0 (0.0)	*		
	Carrying goods or people on back	Yes	0.26 ± 0.10	0.0 (1.0)	3.06 (1.50,6.27)	6.82	**0.009**
		No	0.10 ± 0.03	0.0 (0.0)	*		
Presence of hoof neglect	Type of farm	Pastoral	0.03 ± 0.02	0.0 (1.0)	17.28 (9.71,30.76)	2.74	0.099
		Arable	0.02 ± 0.05	0.0 (0.0)	*		
		Mixed	0.10 ± 0.04	0.0 (1.0)	7.14 (3.84,13.28)		
	Type of bit	“Humane” metal bit	0.17 ± 0.03	0.0 (1.0)	0.14 (0.08,0.27)	3.29	**0.011**
		“Traumatic” metal bit	0.66 ± 0.01	1.0 (0.0)	3.37 (1.54,7.39)		
		Rope bit	0.73 ± 0.05	1.0 (0.0)	0.64 (0.28,1.47)		
		No bit	0.76 ± 0.04	1.0 (0.0)	*		
	Used for draught work	Yes	0.11 ± 0.01	1.0 (1.0)	0.80 (0.48,1.32)	5.15	**0.024**
		No	0.01 ± 0.00	1.0 (1.0)	*		
	Used for breeding/other work	Yes	0.06 ± 0.01	1.0 (1.0)	5.12 (3.20,8.14)	3.35	0.068
		No	0.02 ± 0.01	1.0 (1.0)	*		
BCS	Type of bit	“Humane” metal bit	4.5 ± 0.1 4 (1)	4.0 (1.0)	0.12 (0.07,0.22)	7.13	**<0.001**
		“Traumatic” metal bit	3.9 ± 0.0 4 (0)	4.0 (0.0)	0.90 (0.52,1.57)		
		Rope bit	4.2 ± 0.2 4 (0.5)	4.0 (0.5)	0.57 (0.27,1.19)		
		No bit	3.9 ± 0.1 4 (0)	4.0 (0.0)	*		

**Table 6:** Results of Generalised Linear Mixed Models (GLMM) models testing showing relationship between animal-based parameters and animal-based outcome measures. Only tendencies (*p* <0.01) or significant effects (*p* < 0.05 in bold) are presented here. The parameter levels where no data was available (e.g., reference level for odds ratio) were identified with an asterisk (*). The means (± standard error of the means), medians, interqurtile ranges (IQR), odds ratio (including associated 95% confidence intervals), F statistics and *p*-values are reported at parameter level.

Animal-Based Outcome Measures	Animal-Based Parameters	Level	Mean ± SE	Median (IQR)	Odds Ratio (95% CI)	F st	*p*-Value
Presence of hoof neglect	Horse age group	<6 years	0.01 ± 0.00	1.0 (1.0)	*	5.30	**0.005**
		6–15 years	0.02 ± 0.01	1.0 (0.0)	2.26 (1.58,3.24)		
		>15 years	0.10 ± 0.02	1.0 (0.0)	9.37 (2.18,40.34)		
	General attitude	Positive behaviour	0.01 ± 0.01	1.0 (1.0)	*	7.31	**0.007**
		Negative behaviour	0.12 ± 0.02	1.0 (0.0)	1.83 (0.96,3.52)		
Presence of wounds	General attitude	Positive behaviour	0.04 ± 0.01	0.0 (0.0)	*	3.44	0.064
		Negative behaviour	0.15 ± 0.02	0.0 (0.0)	0.90 (0.48,1.68)		
	Hoof neglect	Present	0.06 ± 0.01	0.0 (0.0)	0.61 (0.40,0.92)	4.30	**0.039**
		Absent	0.13 ± 0.02	0.0 (1.0)	*		
BCS	Hoof neglect	Present	3.90 ± 0.1 4 (0)	4.0 (0.0)	10.79 (7.21,16.14)	7.83	**0.005**
		Absent	4.60 ± 0.1 5 (1)	5.0 (1.0)	*		

## Supplementary Materials

The following are available online at https://www.mdpi.com/2076-2615/10/3/392/s1, Table S1: Owner questionnaire, Table S2: Horse form.
