# Supplementary material for: Welfare Assessment and Husbandry Practices of Working Horses in Fiji
Source: Animals (Basel). 2020 Feb 28;10(3):392. doi: 10.3390/ani10030392 (PMC7142562; doi:10.3390/ani10030392)
Supplement: Supplementary file 1 [file animals-10-00392-s001.pdf]

Article

Supplementary Materials: Welfare Assessment and Husbandry Practices of Working Horses in Fiji

Navina Fröhlich [1,*], Patrick D. Sells [2], Rebecca Sommerville [3], Charlotte F. Bolwell [4], Charlotte Cantley [5], Jessica E. Martin [6,*], Stuart J. G. Gordon [4] and Tamsin Coombs [7]

[1] The Royal (Dick) School of Veterinary Studies, The College of Medicine and Veterinary Medicine, Easter Bush Campus, The University of Edinburgh, Edinburgh, EH25 9RG, UK; Navina.Froehlich@protonmail.com
[2] Chasemore Farm, Cobham, Surrey, KT11 3JT UK; pat@chasemorefarm.co.uk
[3] Brooke, 2nd Floor, The Hallmark Building, 52–56 Leadenhall Street, London, EC3M 5JE, UK; Becca-S@hotmail.co.uk
[4] School of Veterinary Science, Massey University, Tennent Drive, Palmerston North 4474, New Zealand; C.Bolwell@massey.ac.nz (C.F.B.); S.J.G.Gordon@massey.ac.nz (S.J.G.G.)
[5] New Zealand Veterinary Association, Level 2, 44 Victoria Street, Wellington 6011, New Zealand; charlotte.cantley@vets.org.nz
[6] The Royal (Dick) School of Veterinary Studies and The Roslin Institute, The College of Medicine and Veterinary Medicine, Easter Bush Campus, The University of Edinburgh, Edinburgh, EH25 9RG, UK; Jessica.Martin@ed.ac.uk
[7] Animal & Veterinary Sciences, Scotland's Rural College (SRUC), Peter Wilson Building, Kings Buildings, West Mains Road, Edinburgh, EH9 3JG., UK; Tamsin.Coombs@sruc.ac.uk
* Correspondence: Navina.Froehlich@protonmail.com (N.F.); Jessica.Martin@ed.ac.uk (J.E.M.)

Received: 7 February 2020; Accepted: 25 February 2020; Published: 28 February 2020

Table S1. Owner questionnaire.

**ANIMALS FIJI & PACIFIC EQUINE EDUCATION TRUST FIJI QUESTIONNAIRE**			
**Section 1 – Location & General Information**			
**a. 1 (Ba Province)**		b. District:	
**2 (Nadroga/Navosa Province)**			
3 (Ra Province)			
c. Village:			
d. Farm GPS co-ordinates:		S	
**(Mark Waypoint on GPS)**		E	
e. Name of animal owner:			
f. Gender	Male	h. Race	Fijian
	Female		Indian-Fijian
g. Age of owner:			
i. Number of dependents in family:			
j. Type of farm:			a. Pastoral
			b. Arable
k. Lease			a. Formal
			b. Informal
Section 2 – Number and types of livestock owned:			

Species	Number
Horses	
Cows	
Chicken	
Goats	
Sheep	
Pigs	

Section 3 – Husbandry:			
	Frequency:	Done by…	
a. Deworming:			
b. Dental work:			
c. Farrier work:			
d. Castration:	i. Age performed:		
	ii. Technique:	Traditional (tied up)	
		Conventional (using drugs, sterile)	
	iii. Who does this:	Untrained local person	
		Trained health officer	
	iv. Reason for castration:	1.Domestication	
		2.Better condition/growth	
		3.Better behaviour/easier handling	
		4.Population control	
		5.Other	

Section 4: Watering	
a. Do the horses have constant access to water?	Yes
	No
b. If no, how many times are they watered per day?	

Table S2. Horse form.

Healthcare		
5a. How long do you think a horse lives?	……………………years	
b. At what age is the horse when you start to ride it?		
c. Who carries out healthcare? (record name and contact details)		
d. How long have you been working with horses?	……………………years	
e. How many horses have died during this period?		
f. What were the cause(s) of death?		
g. What do you do when the horse is reaching the end of its working life and can no longer work?	Let it go	
	Hanging	
	Other	
h. What do you think are the most important health problems in your horses?		
i. How do you try to solve these problems?		
6a. What do you think are the most important health problems in your cattle?		
b. How do you try to solve these problems?		

7a. Have you heard of Kiwi Care Team before?	Yes	
	No	
b. Have you heard of Animals Fiji before?	Yes	
	No	
c. Have you had animals treated by KCT before?	Yes	
	No	
d. Have you had animals treated by Animals Fiji before?	Yes	
	No	
e. Are there any services you would like KCT to provide for your horse?	1. Castration	
	2. Hoof care	
	3. Teeth care	
	4. Wound care	
	5. Other	
f. Would you still use KCT/AnimalsFiji services if a small fee was charged?	Yes	
	No	
g. If so how much would you be willing to pay for a treatment?	FJD	
h. Do you want veterinary health and animal husbandry workshops to be run by KCT within the community?	Yes	
	No	
	Day(s)	

i. If so, how often, and during which **days/times** would you prefer to attend?	Time(am/pm)		
	Frequency	1/yr	
		2/yr	
		3/yr	

8a. Do you use any medicinal plants on your animals?	Yes	
	No	
b. If so, which plants, and for what conditions?		

Supplementary Table S2: Horse form

Horse Owner:	a. Number (1/2/3 etc.)		
	b. Colour	Bay	
		Chestnut	
		Black	
		Grey	
		Other	
	c. Age (according to owner)		

d. Gender:	1.Filly/mare	
	2.Colt/stallion	
	3.Gelding	
e. Body Condition Score:	1	
	2	
	3	
	4	
	5	
	6	
	7	
	8	
	9	
f. Bit type:	1.No bit	
	2.Rope	
	3.Metal (traumatic)	
	4.Metal (humane)	
	5.Other (describe)	
g. Are there signs of hoof neglect?	Yes	
	No	

h. Lip lesions:	0	
	1	
	2	
	3	
i. Use(s): Ask owner<br><br>Describe ‘Other’ here:	1.Transport (cart)	
	2. Transport (pack)	
	3. Tourist riding	
	4. Riding (owner)	
	5. Agriculture/ploughing	
	6. Foal	
	7. Hunting	
	8. Fishing	
	9. Racing	
	10. Breeding/Trading	
	11. Other	
j. Number of hours used/day		
k. Number of days used/week		
l. General attitude:	0 (Alert, friendly, relaxed)	
	1 (Dull, depressed, sick-looking)	
	2 (Nervous, aggressive)	

m. Does the horse have any swollen joints?	Yes		n. Location of swollen joints (& number):	1. Fetlock	
	No			2. Hock	
				3. Knee	

o. Wound map, excluding lips (mark location):

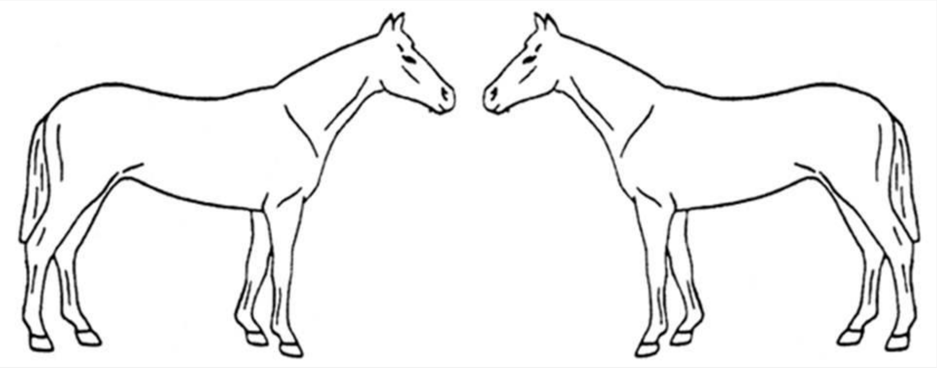

Wound number	Score (1-3)	Approx surface area (cm x cm)	Signs of infection (Y/N)
1			
2			
3			
4			
